# Publisher Correction: Genetic dissection of down syndrome‑associated alterations in APP/amyloid‑β biology using mouse models

**DOI:** 10.1038/s41598-021-94313-2

**Published:** 2021-07-16

**Authors:** Justin L. Tosh, Elena R. Rhymes, Paige Mumford, Heather T. Whittaker, Laura J. Pulford, Sue J. Noy, Karen Cleverley, LonDownS Consortium, Andre Strydom, Elizabeth Fisher, Frances Wiseman, Dean Nizetic, John Hardy, Victor Tybulewicz, Annette Karmiloff-Smith, Matthew C. Walker, Victor L. J. Tybulewicz, Rob C. Wykes, Elizabeth M. C. Fisher, Frances K. Wiseman

**Affiliations:** 1 Department of Neuromuscular Diseases, Queen Square Institute of Neurology, University College London, Queen Square, London, WC1N 3BG UK; 2 The Francis Crick Institute, London, NW1 1AT UK; 3 The UK Dementia Research Institute, University College London, Queen Square, London, WC1N 3BG UK; 4 Department of Clinical and Experimental Epilepsy, Institute of Neurology, University College London, Queen Square, London, WC1N 3BG UK; 5 Department of Immunology and Inflammation, Imperial College, London, W12 0NN UK; 6 Nanomedicine Lab and the Geoffrey Jefferson Brain Research Center, University of Manchester, Manchester, M13 9PT UK; 7 Department of Forensic and Neurodevelopmental Sciences, Institute of Psychiatry, Psychology and Neuroscience, King’s College London, London, UK; 8 Division of Psychiatry, University College London, London, UK; 9 Blizard Institute, Barts and the London School of Medicine, Queen Mary University of London, London, UK; 10 Lee Kong Chian School of Medicine, Nanyang Technological University, Singapore, Singapore; 11 Reta Lila Weston Institute, Institute of Neurology, University College London, London, UK; 12 Birkbeck University, London, UK

Correction to: *Scientific Reports* https://doi.org/10.1038/s41598-021-85062-3, published online 11 March 2021

The original version of this Article contained errors in the Affiliations of the LonDownS Consortium group.

Andre Strydom, Elizabeth Fisher, Frances Wiseman, Dean Nizetic, John Hardy and Victor Tybulewicz were incorrectly affiliated with ‘Birkbeck University, London, UK.’

In addition, Annette Karmiloff‑Smith was incorrectly affiliated with ‘Lee Kong Chian School of Medicine, Nanyang Technological University, Singapore, Singapore.’

The correct affiliations are listed below:

Affiliation 1:

Department of Neuromuscular Diseases, Queen Square Institute of Neurology, University College London, Queen Square, London, UK.

Elizabeth Fisher

Affiliation 2:

The Francis Crick Institute, London, UK.

Victor L. J. Tybulewicz

Affiliation 3:

The UK Dementia Research Institute, University College London, Queen Square, London, UK.

Frances K. Wiseman

John Hardy

Affiliation 5:

Department of Immunology and Inflammation, Imperial College, London, UK.

Victor L. J. Tybulewicz

Affiliations 7 and 8:

Department of Forensic and Neurodevelopmental Sciences, Institute of Psychiatry, Psychology and Neuroscience, King’s College London, London, UK.

Division of Psychiatry, University College London, London, UK.

Andre Strydom

Affiliations 9 and 10:

Blizard Institute, Barts and the London School of Medicine, Queen Mary University of London, London, UK.

Lee Kong Chian School of Medicine, Nanyang Technological University, Singapore, Singapore.

Dean Nizetic

Affiliation 11:

Reta Lila Weston Institute, Institute of Neurology, University College London, London, UK.

John Hardy

Affiliation 12:

Birkbeck University, London, UK.

Annette Karmiloff-Smith

The original Article has been corrected.

