# Supplementary material for: Genetic dissection of down syndrome-associated alterations in APP/amyloid-β biology using mouse models
Source: Sci Rep. 2021 Mar 11;11:5736. doi: 10.1038/s41598-021-85062-3 (PMC7952899; doi:10.1038/s41598-021-85062-3)
Supplement: Supplementary file 1 — Supplementary Information 1. [file 41598_2021_85062_MOESM1_ESM.docx]

**Supplementary material**

**Genetic dissection of Down syndrome-associated alterations in APP/amyloid-β biology using mouse models**

Justin L. Tosh^1,2^, Elena R. Rhymes^1^, Paige Mumford^3^, Heather T. Whittaker^1^, Laura J. Pulford^1^, Sue J. Noy^1,4^, Karen Cleverley^1,4^, LonDownS Consortium^4^, Matthew C. Walker^5^, Victor L.J. Tybulewicz^2,4,6^, Rob C. Wykes^5,7^, Elizabeth M.C Fisher^1,4¶^, Frances K. Wiseman^3,4¶^

**Fig. S1**

**
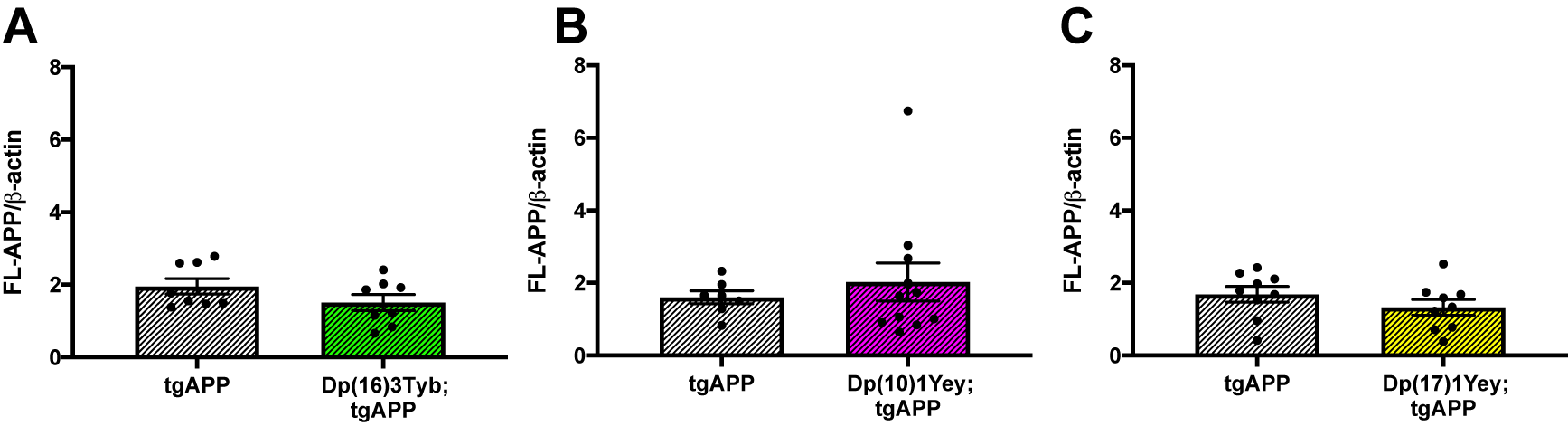
**

**Fig. S1 Abundance of FL-APP is not affected by duplications in the DS mouse models at 3-months of age in the cortex.**

The abundance of full-length APP (FL-APP) relative to β-actin loading control was measured by western blot using A8717 primary antibody in the cortex at 3-months of age in male and female mice. **(A)** There was no difference in FL-APP level between Dp(16)3Tyb;tgAPP (n = 8, 4 male and 4 female) and tgAPP (n = 8, 4 male and 4 female) littermate controls (F(1,12) = 1.896, p = 0.194)**. (B)** No difference in FL-APP level between Dp(10)1Yey;tgAPP (n = 11, 7 male and 4 female) and tgAPP (n = 7, 3 male and 4 female) littermate controls (F(1,14) = 0.520 , p = 0.576). **(C)** No difference in FL-APP level between Dp(17)1Yey;tgAPP mice (n = 9, 6 male and 3 female) and tgAPP littermate controls (F(1,14) = 0.500, p = 0.491). Error bars show SEM, data points are independent mice.

**Fig. S2**


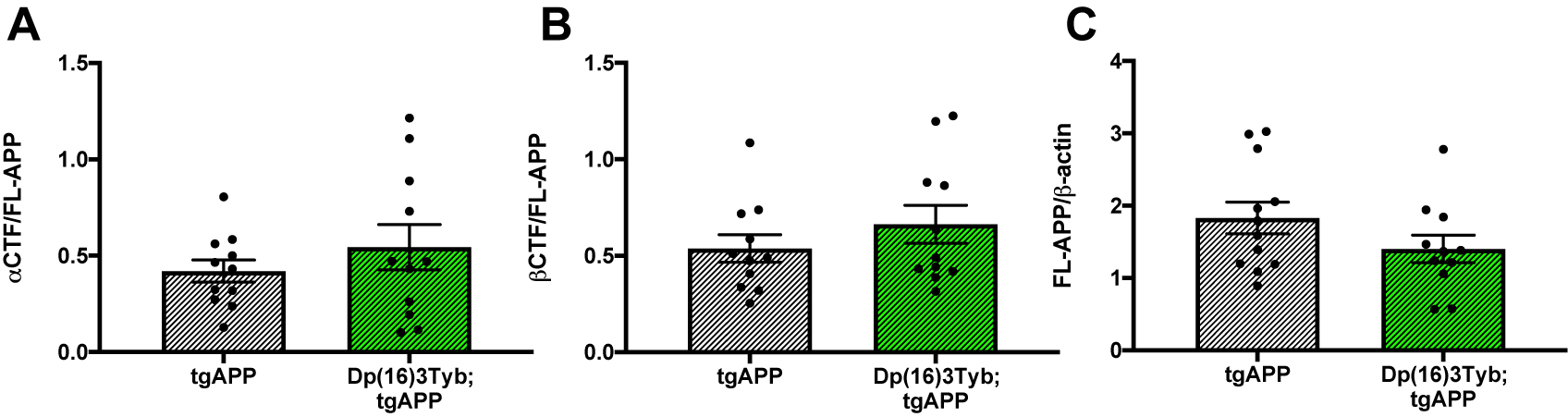


**Fig. S2 Abundance of FL-APP and CTFs is not altered by the Dp(16)3Tyb duplication at 3-months of age in the hippocampus.**

The abundance of full-length APP (FL-APP) relative to β-actin loading control, and APP β-C-terminal fragment (β-CTF) and APP α-C-terminal fragment (α-CTF) relative to full-length APP (FL-APP) was measured by western blot using A8717 primary antibody in the hippocampus at 3-months of age in female and male mice. No difference in, or **(A)** α-CTF (F(1,10) = 0.019, p = 0.892) **(B)** β-CTF (F(1,11)= 1.493, p = 0.247) **(C)** FL-APP (F(1,11) = 1.305, p = 0.277) abundance between Dp(16)3Tyb;tgAPP (n = 12, male = 8 and female = 4) and tgAPP (n = 12, male = 8 and female = 4) littermate controls. Error bars show SEM, data points are independent mice.

**Fig. S3**

**
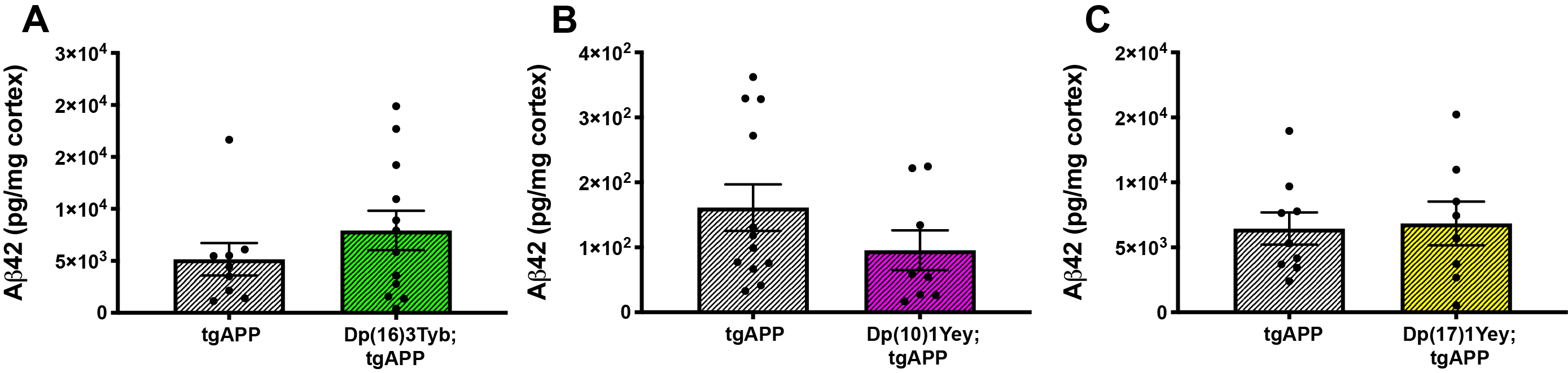
**

**Fig. S3 An additional copy of Hsa-21 homologues from the Dp(16)3Tyb, Dp(10)1Yey, or Dp(17)1Yey regions did not alter the abundance of insoluble amyloid-β_42_ in the cortex at 6-months of age**.

1. In Dp(16)3Tyb;tgAPP mice (n = 12, 7 male and 5 female) insoluble amyloid-β_42_ abundance (F(1,16) = 1.851 p = 0.192) did not significantly differ from tgAPP (n = 10, 3 male and 7 female) littermates at 6-months of age.
2. In Dp(10)1Yey;tgAPP (n = 8, 3 male and 5 female) mice insoluble amyloid-β_42_ abundance (F(1,14) = 2.990 p = 0.106) did not significantly differ from tgAPP (n = 12, 7 male and 5 female) littermates at 6-months of age.
3. In Dp(17)1Yey;tgAPP (n = 8, 4 male and 4 female) mice insoluble amyloid-β_42_ abundance (F(1,11) = 0.299 p = 0.596) did not significantly differ from tgAPP (n = 9, 4 male and 5 female) littermates at 6-months of age.

Error bars show SEM, data points are independent mice.


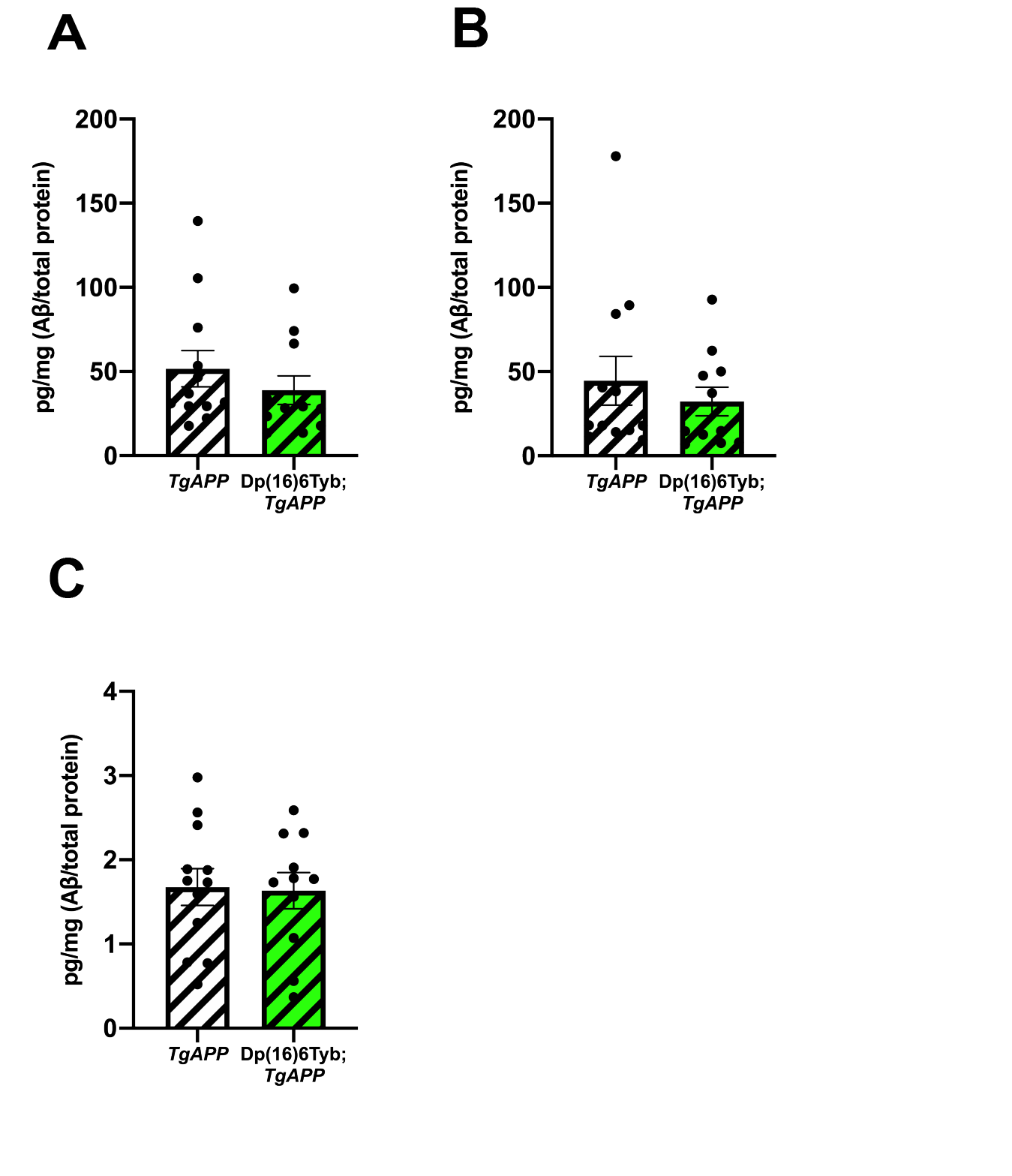

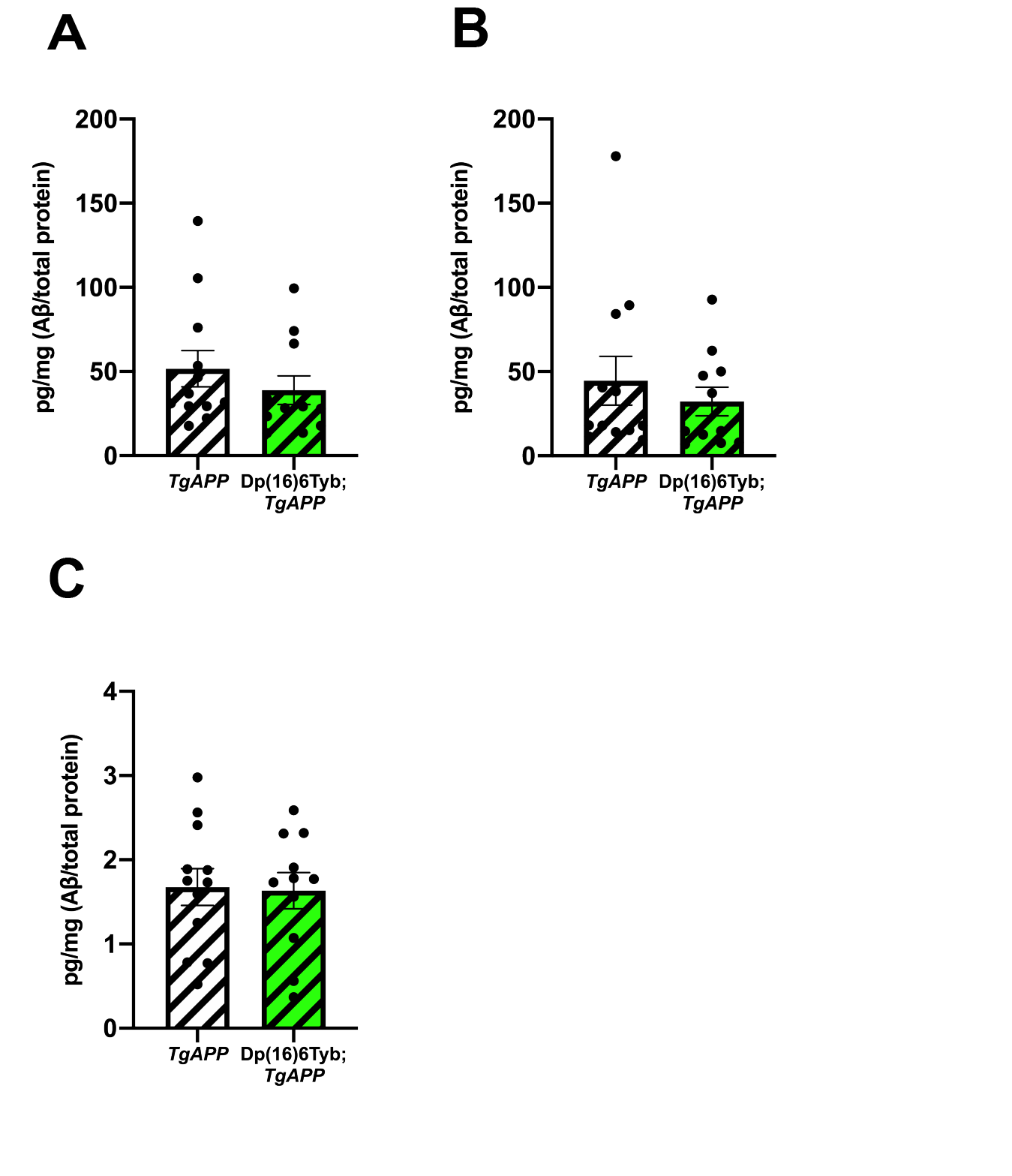


**Fig S4 An additional copy of Hsa-21 homologues from the Dp(16)3Tyb region did not alter the abundance of soluble amyloid-β_40_, amyloid-β_42_, or amyloid-β_40/42_ in the hippocampus at 3-months of age**.

1. In Dp(16)3Tyb;tgAPP mice (n = 12, 8 male and 4 female) soluble amyloid-β_40_ abundance in the hippocampus (F(1,19) = 0.119 p = 0.734) did not significantly differ from tgAPP (n = 11, 6 male and 5 female) littermates at 3-months of age.
2. In Dp(16)3Tyb;tgAPP mice (n = 12, 8 male and 4 female) soluble amyloid-β_42_ abundance in the hippocampus (F(1,19) = 0.012 p = 0.914) did not significantly differ from tgAPP (n = 11, 6 male and 5 female) littermates at 3-months of age.
3. In Dp(16)3Tyb;tgAPP mice (n = 12, 8 male and 4 female) soluble amyloid-β_40/42_ ratio in the hippocampus (F(1,19) = 0.140 p = 0.713) did not significantly differ from tgAPP (n = 11, 6 male and 5 female) littermates at 3-months of age.

Error bars show SEM, data points are independent mice.

**Fig. S5**

**
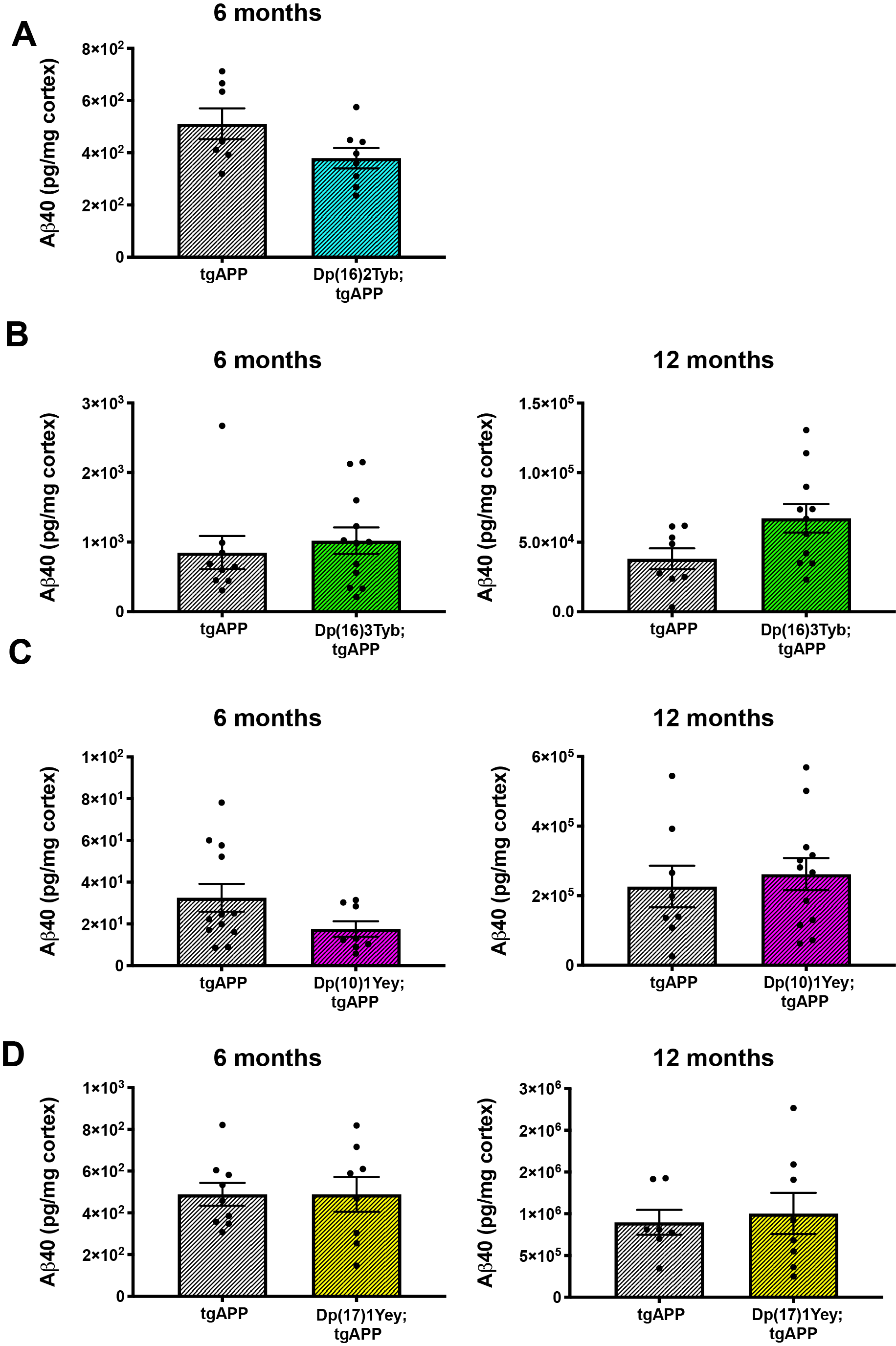
**

**Fig. S5 An additional copy of Hsa-21 homologues from the Dp(16)2Tyb, Dp(16)3Tyb, Dp(10)1Yey, or Dp(17)1Yey regions did not alter the abundance of insoluble amyloid-β_40_ in cortex at 6- or 12- months of age**.

1. In Dp(16)2Tyb;tgAPP (n = 8, 3 male and 5 female) mice insoluble amyloid-β_40_ abundance (F(1,9) = 0.3.739 p = 0.085) did not significantly differ from tgAPP (n = 7, 2 male and 5 female) littermates at 6-months of age.
2. In Dp(16)3Tyb;tgAPP (n = 12, 7 male and 5 female) mice insoluble amyloid-β_40_ abundance (F(1,16) = 0.654 p = 0.431) did not significantly differ from tgAPP (n = 10, 3 male and 7 female) littermates at 6-months of age. In Dp(16)3Tyb;tgAPP mice (n = 11, 7 male and 4 female) insoluble amyloid-β_40_ abundance (F(1,13) = 2.776, p = 0.120) did not significantly differ from tgAPP (n = 8, 5 male and 3 female) littermates at 12-months of age .
3. In Dp(10)1Yey;tgAPP (n = 8, 3 male and 5 female) mice insoluble amyloid-β_40_ abundance (F(1,14) = 3.417 p = 0.086) did not significantly differ from tgAPP (n = 12, 7 male and 5 female) littermates at 6-months of age. In Dp(10)1Yey;tgAPP (n = 12, 6 male and 6 female) mice insoluble amyloid-β_40_ abundance (F(1,14) = 1.112, p = 0.307) did not significantly differ from tgAPP (n = 8, 5 male and 3 female) littermates at 12-months of age.
4. In Dp(17)1Yey;tgAPP (n = 8, 4 male and 4 female) mice insoluble amyloid-β_40_ abundance (F(1,11) = 0.498, p = 0.495) did not significantly differ from tgAPP (n = 9, 4 male and 5 female) littermates at 6-months of age. In Dp(17)1Yey;tgAPP (n = 8, 3 male and 5 female) mice insoluble amyloid-β_40_ abundance (F(1,9) = 0.645, p = 0.443) did not significantly differ from tgAPP (n = 7, 4 male and 3 female) littermates at 12-months of age.

Error bars show SEM, data points are independent mice.

**Fig. S6**

**
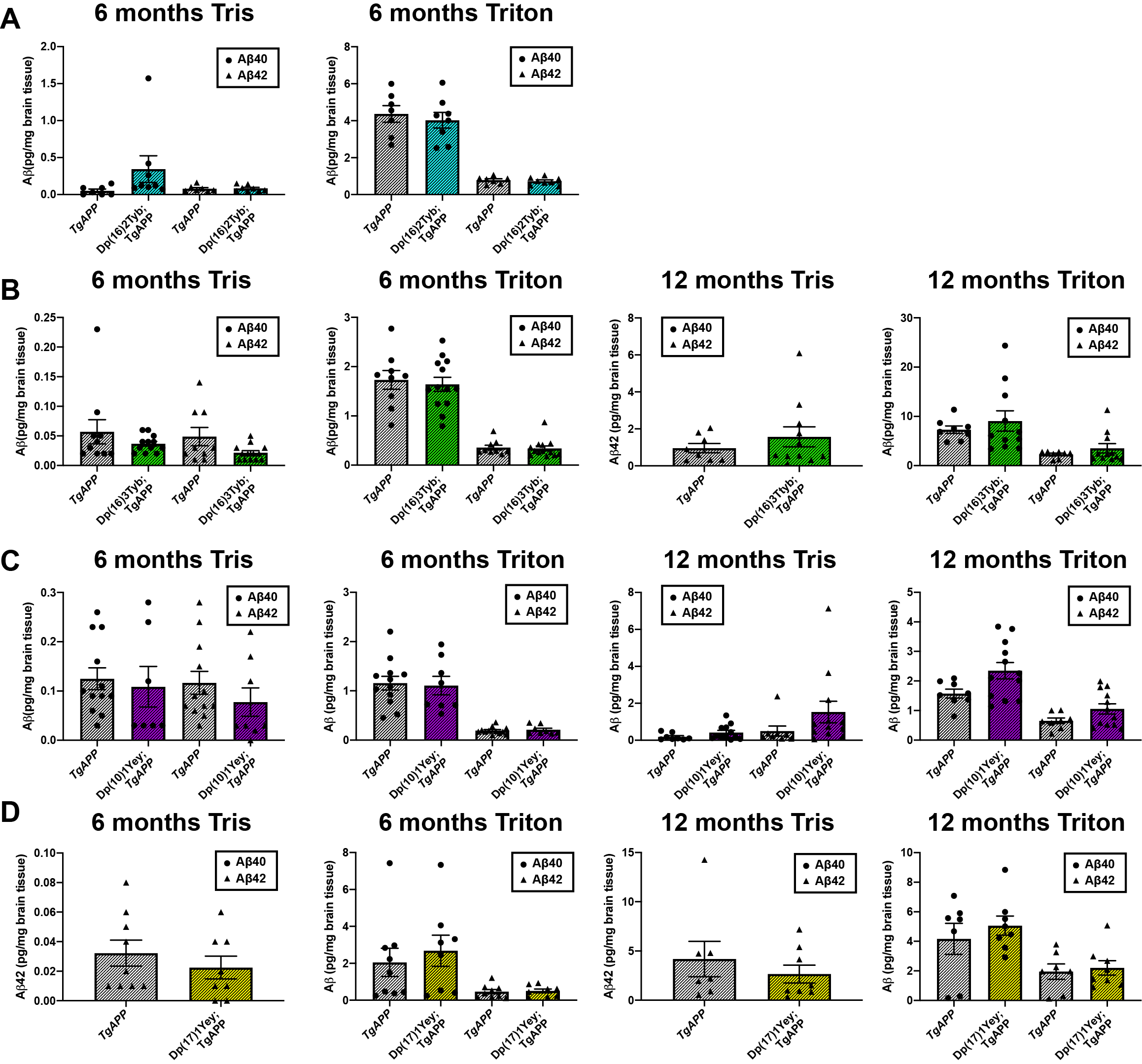
**

**Fig. S6 The effect of an additional copy of Hsa-21 homologues from the Dp(16)2Tyb, Dp(16)3Tyb, Dp(10)1Yey, or Dp(17)1Yey regions on soluble Tris and Triton amyloid-β_40_ and amyloid-β_42_ in the cortex at 6- or 12- months of age**.

1. In Dp(16)2Tyb;tgAPP mice in the soluble Tris fraction, amyloid-β_4o_ abundance (F(1,9) = 1.131, p = 0.315) and amyloid-β_42_ abundance (F(1,9) = 0.261, p = 0.622) did not significantly differ from tgAPP littermates at 6-months of age. In Dp(16)2Tyb;tgAPP mice in the soluble Triton fraction, amyloid-β_4o_ abundance (F(1,9) = 0.08, p = 0.784) and amyloid-β_42_ abundance (F(1,9) = 0.224, p = 0.647) did not significantly differ from tgAPP littermates at 6-months of age. Dp(16)2Tyb;tgAPP (n = 8, 3 male and 5 female) tgAPP (n = 7, 2 male and 5 female).
2. In Dp(16)3Tyb;tgAPP mice in the soluble Tris fraction, amyloid-β_4o_ abundance (F(1,16) = 0.789, p = 0.387) and amyloid-β_42_ abundance (U(N_Dp(16)3Tyb;tgAPP_ = 12, N_tgAPP_ = 10,) = 44, p = 0.341) did not significantly differ from tgAPP littermates at 6-months of age. In Dp(16)3Tyb;tgAPP mice in the soluble Triton fraction, amyloid-β_4o_ abundance (F(1,16) = 0.006, p = 0.940) and amyloid-β_42_ abundance (F(1,16) = 0.006, p = 0.844) did not significantly differ from tgAPP littermates at 6-months of age Dp(16)3Tyb;tgAPP (n = 12, 7 male and 5 female) tgAPP (n = 10, 3 male and 7 female). In Dp(16)3Tyb;tgAPP mice in the soluble Tris fraction, amyloid-β_42_ abundance (F(1,13) = 0.332, p = 0.574) did not significantly differ from tgAPP littermates at 12-months of age. Amyloid-β_4o_ was below the limit of detection. In Dp(16)3Tyb;tgAPP mice in the soluble Triton fraction, amyloid-β_4o_ abundance (F(1,13) = 0.044, p = 0.837) and amyloid-β_42_ abundance (F(1,13) = 0.352, p = 0.563) did not significantly differ from tgAPP littermates at 12-months of age. Dp(16)3Tyb;tgAPP mice (n = 11, 7 male and 4 female) tgAPP (n = 8, 5 male and 3 female).
3. In Dp(10)1Yey;tgAPP mice in the soluble Tris fraction, amyloid-β_4o_ abundance (F(1,14) = 0.0003, p = 0.986) and amyloid-β_42_ abundance (F(1,14) = 0.256, p = 0.621) did not significantly differ from tgAPP littermates at 6-months of age. In Dp(10)1Yey;tgAPP mice in the soluble Triton fraction, amyloid-β_4o_ abundance (F(1,14) = 0.147, p = 0.707) and amyloid-β_42_ abundance (F(1,14) = 0.559, p = 0.467) did not significantly differ from tgAPP littermates at 6-months of age. Dp(10)1Yey;tgAPP (n = 8, 3 male and 5 female) tgAPP (n = 12, 7 male and 5 female). In Dp(10)1Yey;tgAPP mice in the soluble Tris fraction, amyloid-β_4o_ abundance (F(1,14) = 1.784, p = 0.203) did not significantly differ from tgAPP littermates at 12-months of age however median amyloid-β_42_ was significantly increased (U(N_Dp(10)1Yey;tgAPP_ = 12, N_tgAPP_ = 8,) = 21, p = 0.039). In Dp(10)1Yey;tgAPP mice in the soluble Triton fraction, amyloid-β_4o_ abundance (F(1,14) = 2.715, p = 0.122) and median amyloid-β_42_ (U(N_Dp(10)1Yey;tgAPP_ = 12, N_tgAPP_ = 8,) = 33, p = 270) did not significantly differ from tgAPP littermates at 12-months of age. Dp(10)1Yey;tgAPP (n = 12, 6 male and 6 female) tgAPP (n = 8, 5 male and 3 female).
4. In Dp(17)1Yey;tgAPP mice in the soluble Tris fraction, amyloid-β_42_ abundance (F(1,11) = 0.237 p = 0.636) did not significantly differ from tgAPP littermates at 6-months of age. Amyloid-β_4o_ was below the limit of detection. Dp(17)1Yey;tgAPP (n = 8, 4 male and 4 female) tgAPP (n = 9, 4 male and 5 female). In Dp(17)1Yey;tgAPP mice in the soluble Triton fraction, amyloid-β_4o_ abundance (F(1,11) = 0.490, p = 0.499) and amyloid-β_42_ abundance (F(1,11) = 0.067, p = 0.800) did not significantly differ from tgAPP littermates at 6-months of age. In Dp(17)1Yey;tgAPP mice in the soluble Tris fraction, amyloid-β_42_ abundance (F(1,9) = 0.215 p = 0.654) did not significantly differ from tgAPP littermates at 12-months of age. Amyloid-β_4o_ was below the limit of detection. In Dp(17)1Yey;tgAPP mice in the soluble Triton fraction, amyloid-β_4o_ abundance (F(1,9) 0.58, p = 0.466) and amyloid-β_42_ abundance (F(1,9) = 0.294, p = 0.601) did not significantly differ from tgAPP littermates at 12-months of age. Dp(17)1Yey;tgAPP (n = 8, 3 male and 5 female) tgAPP (n = 7, 4 male and 3 female). Error bars show SEM, data points are independent mice.

**Fig. S7**

**6 months**

**
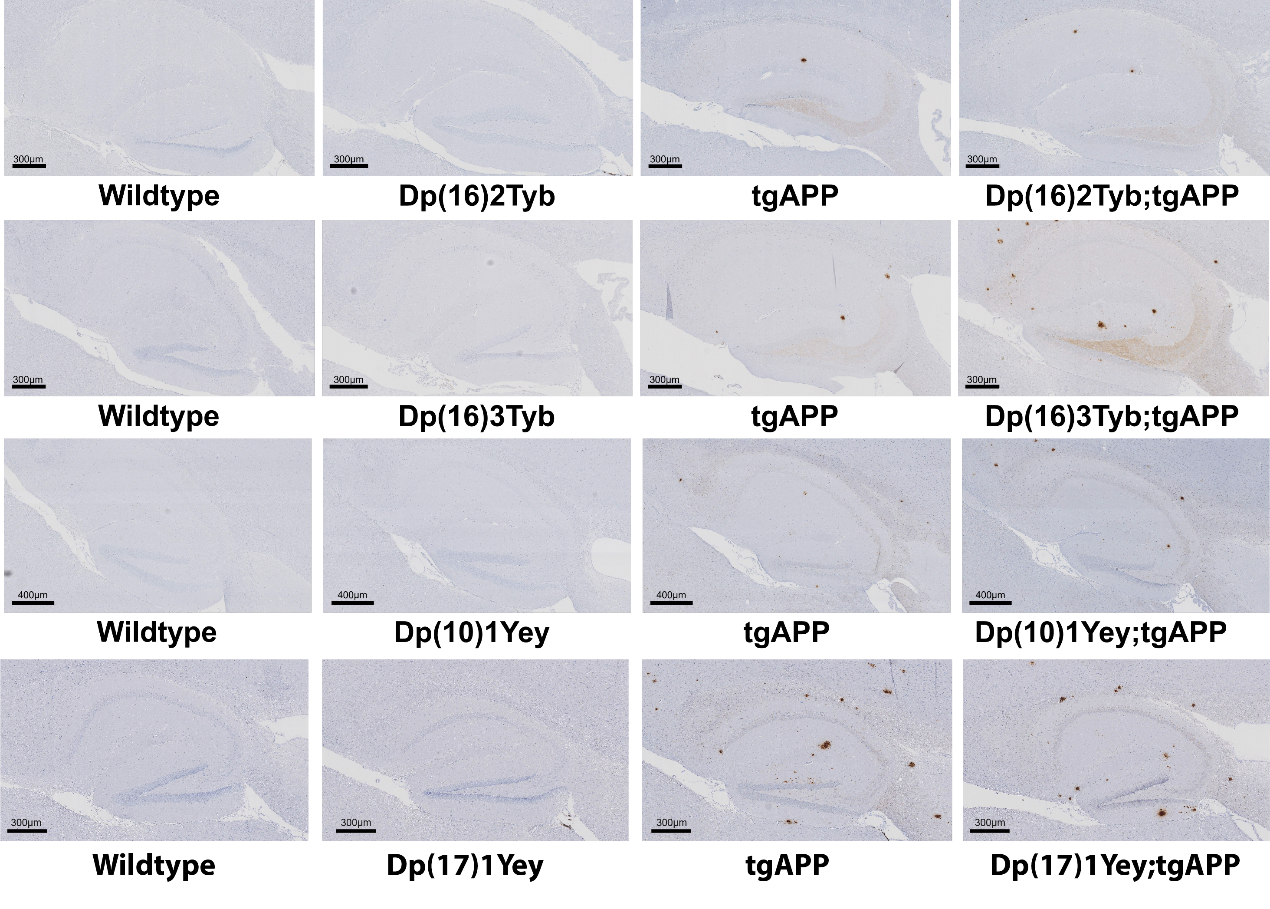
**

**D**

**Cv**

**Av**

**Bv**

**12 months**


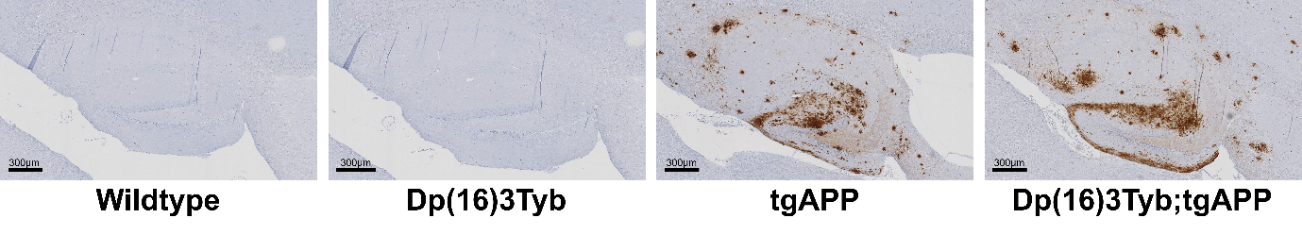


**Ev**


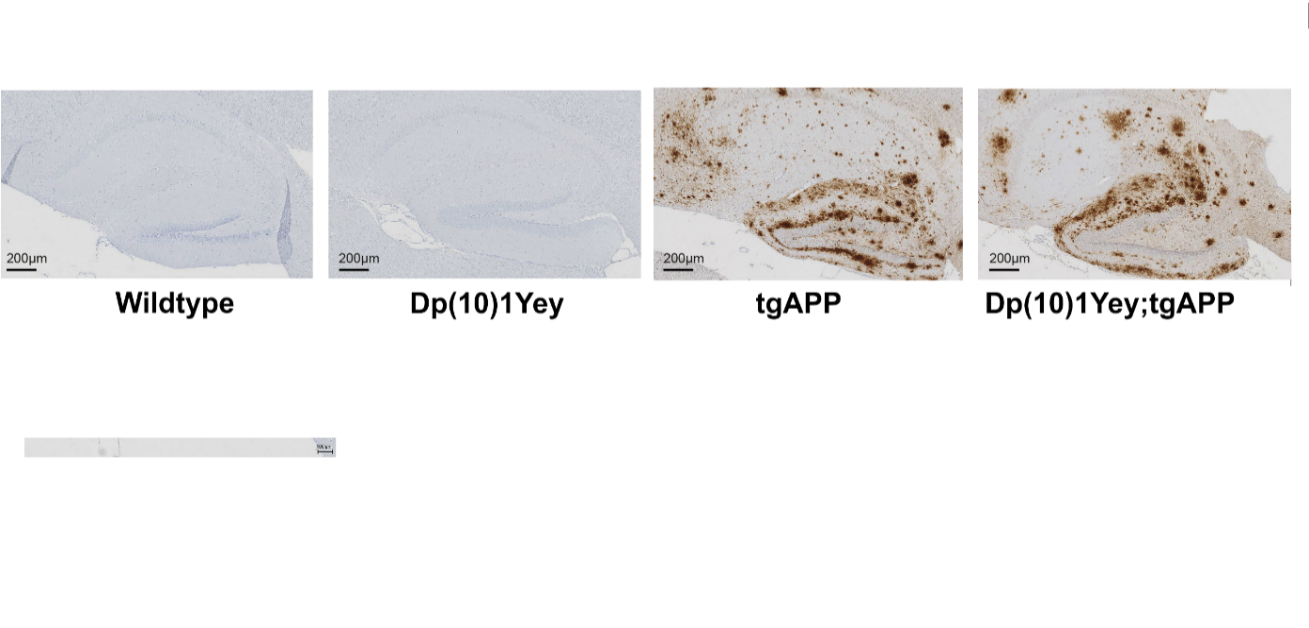


**Fv**


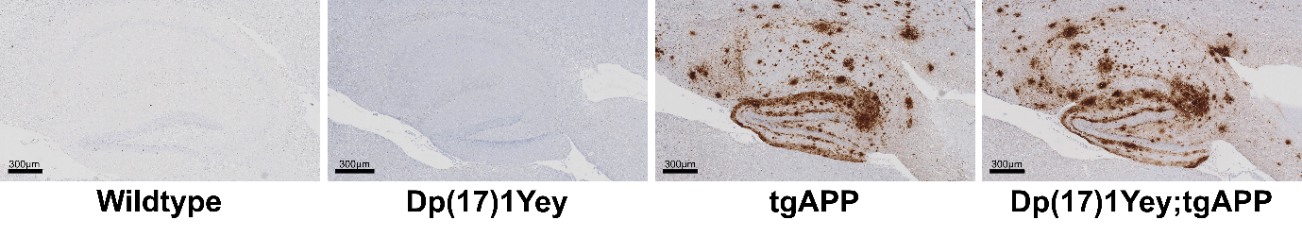


**Gv**

**Fig. S7 Representative images of hippocampus at 12-months of age from the Dp(16)2Tyb, Dp(16)3Tyb, Dp(10)1Yey and Dp(17)1Yey tgAPP crosses (corresponding to the data in Figure 5).**

1. Wildtype, Dp(16)2Tyb, tgAPP and Dp(16)2Tyb;tgAPP at 6-months of age stained with 82E1 primary antibody.
2. Wildtype, Dp(16)3Tyb, tgAPP and Dp(16)3Tyb;tgAPP at 6-months of age stained with 82E1 primary antibody.
3. Wildtype, Dp(10)1Yey, tgAPP and Dp(10)1Yey;tgAPP at 6-months of age stained with 4G8 primary antibody.
4. Wildtype, Dp(17)1Yey, tgAPP and Dp(17)1Yey;tgAPP at 6-months of age stained with 4G8 primary antibody.
5. Wildtype, Dp(16)3Tyb, tgAPP and Dp(16)3Tyb;tgAPP at 12-months of age stained with 82E1 primary antibody
6. Wildtype, Dp(10)1Yey, tgAPP and Dp(10)1Yey;tgAPP at 12-months of age stained with 4G8 primary antibody.
7. Wildtype, Dp(17)1Yey, tgAPP and Dp(17)1Yey;tgAPP at 12-months of age stained with 4G8 primary antibody.

**Fig. S8**

**
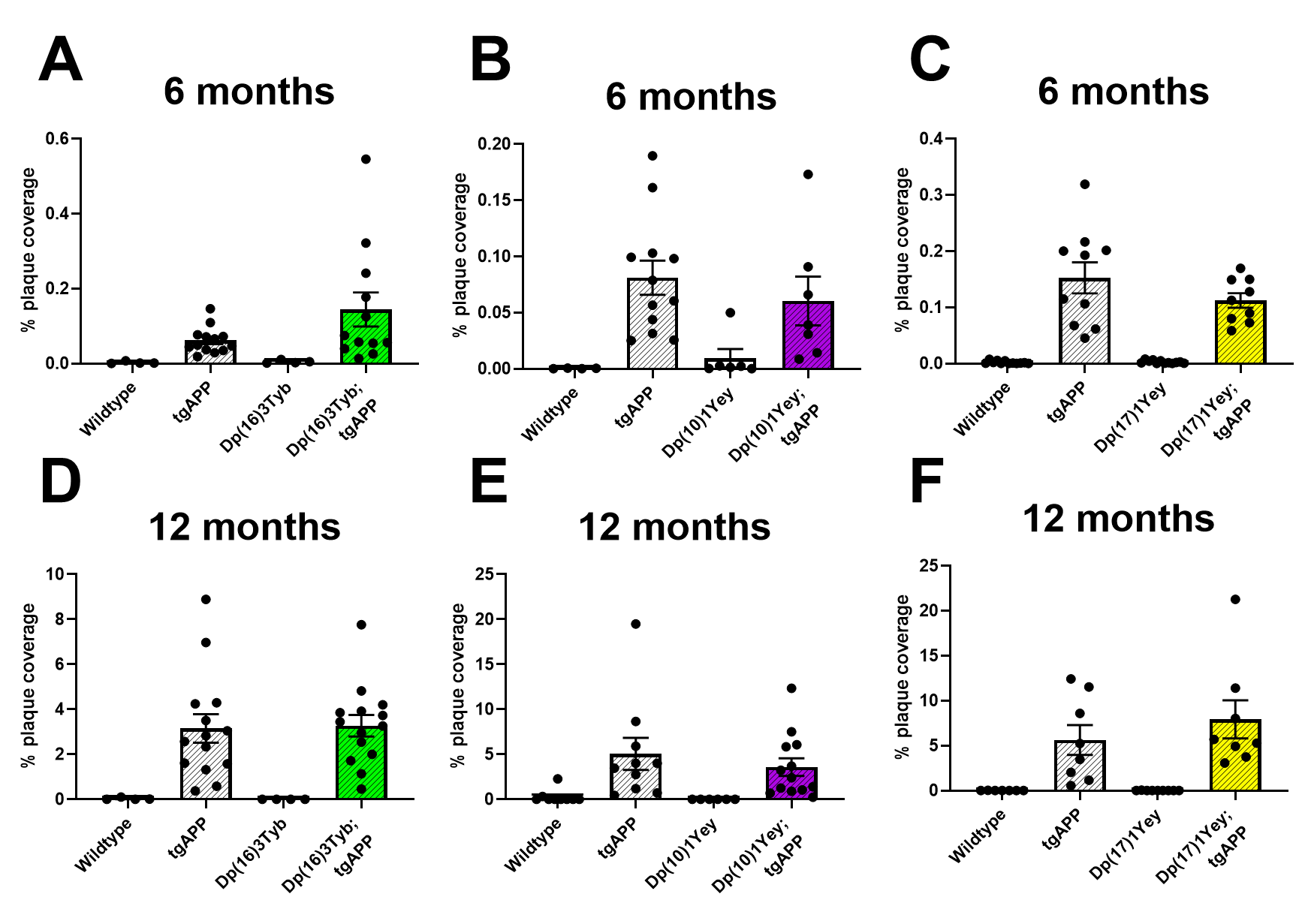
**

**Fig. S8 Deposition of amyloid-β in the cortex at 6- and 12-months of age in the Dp(16)3Tyb, Dp(10)1Yey or Dp(17)1Yey tgAPP double mutants.**

Amyloid-β deposition in the cortex was quantified at (**A-C**) 6- and (**D-F**) 12-months of age in male and female mice, percentage of the region covered by stain was calculated. Error bars show SEM, data points are independent mice.

1. No significant difference in amyloid-β deposition in the cortex was detected at 6-months of age in Dp(16)3Tyb;tgAPP compared with tgAPP controls (*U*(N_Dp(16)3Tyb;tgAPP_ = 12, N_tgAPP_ = 13,) = 56, p = 0.247). After systematic outlier testing one tgAPP sample was excluded prior to analysis. Dp(16)3Tyb;tgAPP female n=6, male n=6; tgAPP female n=7, male n=6.
2. No significant difference in amyloid-β deposition in the cortex was detected at 6-months of age in Dp(10)1Yey;tgAPP compared with tgAPP controls (F(1,14) = 0.246, p = 0.628). Dp(10)1Yey;tgAPP female n=4, male n=3; tgAPP female n=5, male n=7.
3. No significant difference in amyloid-β deposition in the cortex was detected at 6-months of age in Dp(17)1Yey;tgAPP compared with tgAPP controls (*U*(N_Dp(17)1Yey;tgAPP_ = 9, N_tgAPP_ = 10,) = 34, p = 0.400). Dp(17)1Yey;tgAPP female n=5, male n=4; tgAPP female n=6, male n=4.
4. No significant difference in amyloid-β deposition in the cortex was detected at 12-months of age in Dp(16)3Tyb;tgAPP compared with tgAPP controls (F(1,23) = 0.031, p = 0.861). Dp(16)3Tyb;tgAPP female n=8, male n=6; tgAPP female n=7, male n=7.
5. No significant difference in amyloid-β deposition in the cortex was detected at 12-months of age in Dp(10)1Yey;tgAPP compared with tgAPP controls (F(1,18) = 0.056, p = 0.815). Dp(10)1Yey;tgAPP female n=7, male n=6; tgAPP female n=6, male n=4.
6. No significant difference in amyloid-β deposition in the cortex was detected at 12-months of age in Dp(17)1Yey;tgAPP compared with tgAPP controls (F(1,11) = 0.218, p = 0.649). Error bars show SEM, data points are independent mice. Dp(17)1Yey;tgAPP female n=5, male n=3; tgAPP female n=5, male n=3.

**Supplementary Table 1**

Summary of gene expression in the 3-month old hippocampus of the Dp(16)1Tyb mouse model of Down syndrome reported in^1^ and comparison of gene content in the Dp(16)2Tyb and Dp(16)3Tyb mouse models as reported in^2^

**References**

1. Ahlfors, H. *et al.* Gene expression dysregulation domains are not a specific feature of Down syndrome. *Nat. Commun.* **10**, 2489 (2019).

2. Lana-Elola, E. *et al.* Genetic dissection of Down syndrome-associated congenital heart defects using a new mouse mapping panel. *Elife* **5**, 1–20 (2016).
